# First Case of Bioterrorism-Related Inhalational Anthrax, Florida, 2001: North Carolina Investigation

**DOI:** 10.3201/eid0810.020389

**Published:** 2002-10

**Authors:** Jean-Marie Maillard, Marc Fischer, Kelly T. McKee, Lou F. Turner, J. Steven Cline

**Affiliations:** * North Carolina Department of Health and Human Services, Raleigh, North Carolina, USA; † Centers for Disease Control and Prevention, Atlanta, Georgia, USA

**Keywords:** *Bacillus anthracis*, anthrax, bioterrorism, epidemiology and surveillance

## Abstract

The index case of inhalational anthrax in October 2001 was in a man who lived and worked in Florida. However, during the 3 days before illness onset, the patient had traveled through North Carolina, raising the possibility that exposure to *Bacillus anthracis* spores could have occurred there. The rapid response in North Carolina included surveillance among hospital intensive-care units, microbiology laboratories, medical examiners, and veterinarians, and site investigations at locations visited by the index patient to identify the naturally occurring or bioterrorism-related source of his exposure.

The index case of inhalational anthrax in October 2001 was in a man who lived and worked in Florida. However, during the 3 days before illness onset, he had traveled through North Carolina, raising the possibility that exposure to *Bacillus anthracis* spores could have occurred there. On October 4, concurrent investigations were initiated in Florida and North Carolina to identify the naturally occurring or bioterrorism-related source of his exposure. In less than a week, investigators isolated *B. anthracis* from the patient’s place of employment in Florida (1,2). However, the history of travel to North Carolina had already resulted in a substantial public health effort in that state. We review the surveillance methods employed during the rapid response in North Carolina and discuss several lessons that may prove instructive for future investigations.

## Methods

### Surveillance Infrastructure

Retrospective syndrome- and laboratory-based surveillance for illnesses compatible with systemic anthrax infection was initiated on October 5 and continued for the 27 days from September 11 to October 6, 2001. Prospective surveillance was begun on October 7 and suspended on October 12. Based on the index patient’s travel route, surveillance was undertaken in all 15 hospitals with intensive-care units (ICUs) in five North Carolina counties (combined population 1,258,980), and four regional referral centers in North Carolina (n=2) and South Carolina (n=2). These 19 hospitals have a total inpatient capacity of 5,720 beds.

A site coordinator, usually an infection control practitioner or hospital epidemiologist, was identified to lead the investigation at each hospital. The site coordinator communicated 1–2 times a day with a public health official designated as county anthrax surveillance officer. County surveillance officers reported cumulative data at daily conference calls with the state anthrax investigation team, which was based at the North Carolina Department of Health and Human Services (DHHS) in Raleigh. The state medical examiner, state veterinarian, and other experts (e.g., infectious disease clinicians) also participated in the daily conference calls to report any unexplained deaths identified in humans or farm animals and provide consultation as needed. Finally, a statewide information campaign was initiated by using electronic mailings to North Carolina health-care professionals and press releases to increase recognition by clinicians, raise public awareness, and provide contact information for any suspected cases.

### Syndrome-Based Surveillance

For the 19 hospitals, investigators identified all patients admitted to the ICU from September 11 to October 7 who had blood or cerebrospinal fluid cultures obtained at the initial encounter. For patients meeting these criteria, the investigation team reviewed medical records to identify a subset of cases with one of four primary clinical syndromes, including fever and 1) severe respiratory disease (i.e., pneumonia or acute respiratory distress syndrome), 2) mediastinitis or mediastinal lymphadenitis, 3) meningitis, or 4) hemorrhagic gastroenteritis. Additional epidemiologic, clinical, and laboratory data were then obtained to define a specific cause of illness for patients with any of these syndromes.

Beginning October 7, hospital site coordinators reviewed emergency department, ICU, and autopsy logs daily to identify patients who died or were admitted with any of the four suspicious clinical syndromes. A standard report form was completed for each suspected case by abstracting the medical chart and, if needed, interviewing the patient’s physician and family. Active suspected cases were maintained on a daily line list until a specific diagnosis or infectious agent had been identified or the possibility of anthrax had been excluded. A decision tree was developed to assist with finding and evaluating suspected cases (Figure).

**Figure:**
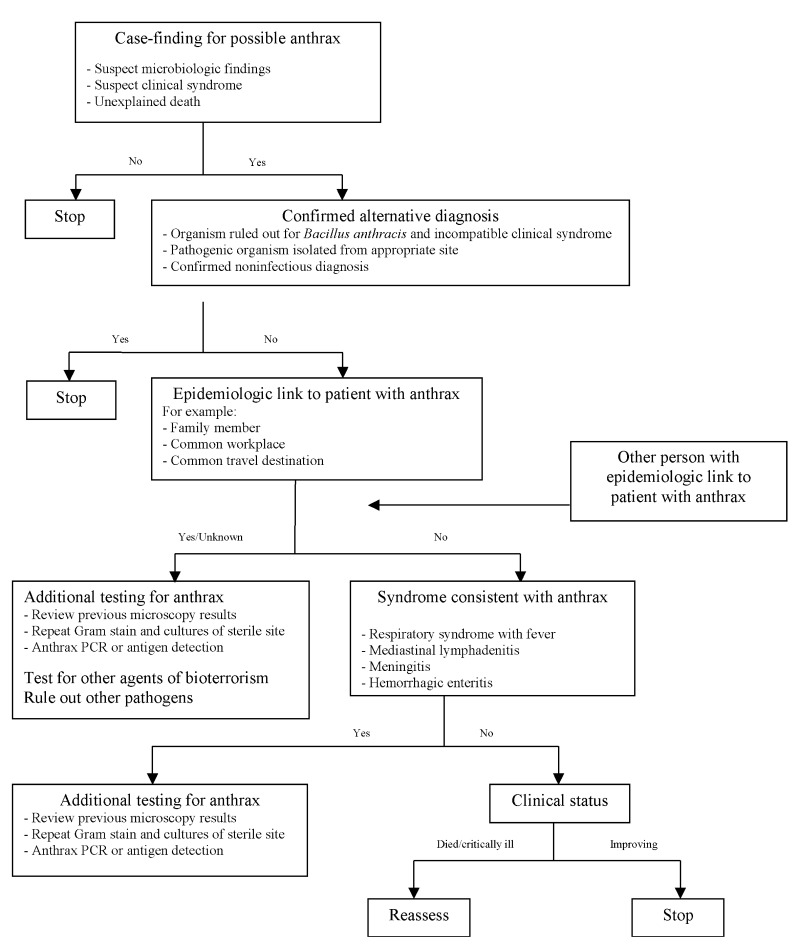
Decision analysis developed during the North Carolina investigation for identifying and evaluating patients with possible systemic anthrax. PCR, polymerase chain reaction.

### Laboratory-Based Surveillance

Microbiology laboratory records from the 19 hospitals were reviewed both retrospectively (from September 11 to October 7) and prospectively (from October 7 to October 12) to identify suspicious bacterial isolates obtained from normally sterile sites (e.g., blood, cerebrospinal fluid, or pleural fluid). A suspicious isolate was defined as 1) nontyped *Bacillus* species, 2) unidentified nonhemolytic, nonmotile gram-positive rod, or 3) any other unidentified isolate that was discarded or sent to a referral laboratory. If the isolate was still available, additional phenotypic testing was performed at a local or reference laboratory to rule out *B. anthracis*. Concurrent with that process or if the isolate had been discarded, the patient’s chart was abstracted to determine if the illness was compatible with systemic anthrax.

### Site Investigation

Two teams of medical epidemiologists, industrial hygienists, and Federal Bureau of Investigation agents surveyed all North Carolina locations the patient visited before illness onset. The environmental investigation focused on two sites, including a relative’s home and a rural tourist park. Family members who stayed or traveled with the index patient were interviewed. Recent illnesses and absences among the 90 employees at the park were reviewed. Available records (e.g., annual pass holders, credit card receipts) for approximately 700 persons who visited the park on the same day as the index patient were held for use in tracking patrons, if needed. Soil, water, vacuum filters, air filters, and swabs of selected surfaces were obtained from both locations to assess for *B. anthracis* spores. Samples were divided for testing at the North Carolina State Laboratory of Public Health and the Centers for Disease Control and Prevention.

## Results

### Syndrome-Based Surveillance

Investigators retrospectively identified 361 patients who were admitted to an ICU from September 11 to October 7 and had blood or cerebrospinal fluid cultures obtained at the initial encounter. Of these, 9 (2%) patients had a clinical syndrome of interest (all fever and severe respiratory disease) and required additional information to rule out a diagnosis of anthrax. The identification of suspected cases through retrospective case finding was completed by the end of the third day of the investigation. During October 7–12, prospective surveillance identified an additional five patients with fever and severe respiratory disease who died or were admitted to an ICU in one of the 19 hospitals under surveillance (Table).

**Table:** Surveillance methods used to identify potential cases of systemic anthrax or a source of exposure for the Florida index case of inhalational anthrax, North Carolina, October 2001

Type of surveillance	Targeted population or outcome	Locations under surveillance
Intensive-care unit	Patients with illness compatible with systemic anthrax infection^a^	19 hospitals in North and South Carolina^b^
Microbiology laboratory	Bacterial isolates potentially consistent with *Bacillus anthracis*^c^	19 hospitals in North and South Carolina
Medical examiner	Unexplained deaths possibly due to anthrax infection	Statewide
Veterinarian	Unexplained deaths in livestock	Statewide
Occupational	Unexplained illnesses or absences in employees	Tourist park visited by the index patient
Environmental	Evidence of *B. anthracis* spores	Residence of index patient’s relative; tourist park visited by the index patient

^a^Clinical syndromes included fever and 1) severe respiratory disease, 2) mediastinitis or mediastinal lymphadenitis, 3) meningitis, or 4) hemorrhagic gastroenteritis. ^b^Based on the index patient’s route of travel, surveillance occurred in all 15 hospitals with intensive-care units in five North Carolina counties, as well as four regional referral centers in North Carolina (n=2) and South Carolina (n=2). ^c^A suspicious isolate was defined as 1) nontyped *Bacillus* species, 2) unidentified nonhemolytic, nonmotile gram-positive rod, or 3) any other unidentified bacteria that was discarded or sent to a referral laboratory.

Of the 14 cases of interest detected through hospital-based retrospective or prospective surveillance, 4 (29%) were fatal. None were due to anthrax. The state medical examiner identified one additional fatal case that warranted further evaluation in a county not included in the surveillance. This case of pneumonia and sepsis in a 10-year-old boy was subsequently attributed to a β-hemolytic streptococcus. No suspicious deaths of animals were reported to the state veterinarian during the relevant time period.

### Laboratory-Based Surveillance

From September 11 to October 12, 10 isolates were identified through hospital microbiology laboratories that required additional investigation. All were either *Bacillus* species that had not undergone further identification or nonspecific gram-positive rods that had not been completely evaluated for hemolysis or motility. None of the patients from whom these bacteria were isolated had clinical courses consistent with inhalational anthrax, and none of the organisms were subsequently identified as *B. anthracis*.

### Site Investigation

No relevant illnesses were identified in close contacts of the index patient in North Carolina or in other patrons or employees of the tourist park. No suspicious events (e.g., aerosol releases) or exposures were identified at any of the locations the patient visited. However, park employees noted that a cow had died of unknown causes in an adjacent orchard approximately 1 year earlier. Although the index patient had not visited this area, he had reportedly drunk water from a stream that traversed the tourist park after passing through the orchard. A total of 35 environmental samples were obtained from sites the index patient visited: 5 (14%) were from the relative’s home and 30 (86%) from the tourist park, including soil from the area where the cow died and water from the stream. Cultures of all environmental specimens were negative for *B. anthracis*.

## Discussion

In 1999, the North Carolina DHHS established short-term hospital-based surveillance in 18 counties to assess injuries and other medical consequences resulting from Hurricane Floyd. This experience was extremely useful in rapidly implementing syndromic surveillance during the anthrax investigation. Nevertheless, limited staffing, absence of electronic surveillance and reporting, the wide geographic area traversed by the patient, intense media scrutiny, and the simultaneous involvement of multiple public health and law enforcement agencies posed major challenges to the investigation.

The North Carolina anthrax investigation team required contributions from many persons of varied expertise, including epidemiologists, microbiologists, pathologists, veterinarians, infectious disease clinicians, infection control practitioners, engineers, industrial hygienists, health communicators, and law enforcement and emergency management personnel. The team operated under a command structure led by the North Carolina Department of Health and Human Services. Participating state and federal agencies were represented at both the investigation headquarters in Raleigh and on each field team. Conference calls that included all decision-making parties were held at the same time each day to rapidly disseminate information throughout team members, and set the specific priorities of the investigation for the next 24 hours. In addition, press releases were distributed regularly to minimize reporting inaccuracies, and dedicated spokespersons were identified to provide a clear and consistent message.

However, several factors could have helped the investigation run more efficiently. First, case definitions, surveillance methods, data collection forms, and informational materials had to be developed ad hoc throughout the investigation, resulting in delays in implementing surveillance, uncertainties as to the effectiveness of and person-hours required by the case-finding methods, and inefficiencies in the data collection process. Second, most of the communications and transfer of information during this investigation occurred by telephone and fax. Although this system was workable given its relatively small scale, it resulted in inefficient data management that would have been rapidly overwhelmed by additional cases or sites. Third, many persons and agencies involved in the investigation had not previously worked together, resulting in a lack of familiarity with their respective organization and capacity. Finally, substantial time and effort were needed during the investigation to educate health-care providers and public health practitioners about the epidemiology and clinical manifestations of inhalational anthrax.

This investigation and its ramifications provided an important learning opportunity and impetus to better prepare for future bioterrorist attacks. Standard protocols, data collection instruments, and informational documents that can be adapted to specific situations are being developed to minimize delays and avoid omissions. In North Carolina, resources are also being used to 1) establish state and regional teams trained in bioterrorism response and 2) develop a statewide Health Alert Network. North Carolina’s network will be a secure multidirectional electronic network through which the state health department can rapidly communicate with hospitals, clinicians, and public health and law enforcement authorities. This new infrastructure will allow for an efficient flow of information during future investigations and provide surge capacity to better respond to requests for assistance at the local level. In addition, health professionals are being educated statewide to better recognize the clinical manifestations of biologic agents that may be used in terrorism. These efforts may build on lessons learned from the fall of 2001 to provide a more rapid, comprehensive, and efficient response to public health emergencies.
